# UV–vis absorbance spectra, molar extinction coefficients and circular dichroism spectra for the two cyanobacterial metabolites anabaenopeptin A and anabaenopeptin B

**DOI:** 10.1016/j.dib.2024.110914

**Published:** 2024-09-15

**Authors:** Till Steiner, Franziska Schanbacher, Wolfram Lorenzen, Heike Enke, Elisabeth M.-L. Janssen, Timo H.J. Niedermeyer, Karl Gademann

**Affiliations:** a Department of Chemistry, University of Zurich, Winterthurerstrasse 190, CH-8057 Zurich, Switzerland; b Institut für Pharmazie, Freie Universität Berlin, Königin-Luise-Strasse 2+4, 14195 Berlin, Germany; c Simris Biologics GmbH, Magnusstrasse 11, 12489 Berlin, Germany; d Swiss Federal Institute of Aquatic Science and Technology (Eawag), Überlandstrasse 133, CH-8600 Düberndorf, Switzerland

**Keywords:** Cyanobacteria, Metabolites, Quantitative NMR, UV–vis spectroscopy

## Abstract

The UV–vis absorbance spectra, molar extinction coefficients and circular dichroism spectra, as well as NMR and high resolution tandem mass spectrometry spectra were determined for two prominent secondary metabolites from cyanobacteria, namely anabaenopeptin A and anabaenopeptin B. The compounds were extracted from the cyanobacterium *Planktothrix rubescens* CBT929 and purified by flash chromatography and HPLC. Exact amounts of isolated compounds were assessed by quantitative ^1^H-NMR with internal calibrant ethyl 4-(dimethylamino)benzoate in DMSO‑*d_6_* at 298 K with a recycle delay (d1) of 120 s. UV–vis absorbance spectra were recorded in methanol at room temperature. Molar extinction coefficients were determined at 278 nm as 4190 M^−1^ cm^−1^ and 2300 M^−1^ cm^−1^ in methanol for anabaenopeptin A and anabaenopeptin B, respectively. Circular dichroism spectra and secondary fragmentation mass spectra are also reported.

Specifications Table

**Table:** 

Subject	Analytical Chemistry: Spectroscopy
Specific subject area	*Spectral properties of anabaenopeptin A and B, two cyanobacterial metabolites*
Type of data	Table, Figure Raw, Analyzed, Processed
Data collection	Anabaenopeptin A and B were extracted from biomass of the cyanobacterium *Planktothrix rubescens* (CBT929), purified by HPLC (Kinetex C18 column), quantified by qNMR (Bruker 500 MHz spectrometer), UV absorbance profiles (Agilent Cary 60) and extinction coefficients as well as circular dichroism spectra (Applied Photophysics Chirascan™) determined and MS^2^ spectra (Thermo Fisher Scientific Q Exactive Plus) recorded.
Data source location	Institution: University of Zurich, Swiss Federal Institute of Aquatic Science and Technology (Eawag), Freie Universität Berlin
Data accessibility	Repository name: UV–vis absorbance data, molar extinction coefficients and circular dichroism spectra for the two cyanobacterial metabolites anabaenopeptin A and anabaenopeptin B [1] Data identification number: 10.5281/zenodo.11203432 Direct URL to data: https://zenodo.org/records/11203432

## 1 Value of Data

• This article includes the first report of UV absorption profile, λ_max_, extinction coefficient (ε; determined using qNMR), and circular dichroism spectra of anabaenopeptin A and B in solvents relevant to detection in biological and/or environmental samples.
• High resolution mass spectrometry data are provided.
• Quantitative NMR was applied for exact quantification of isolated metabolites.
• Data will assist in the quantification of these anabaenopeptins in biological/environmental samples, in the verification of the concentration of bioreagents and reference standards, and in monitoring the stability of stock solutions.

## 2 Background

Anabaenopeptins are non-ribosomal peptides produced by certain cyanobacteria in surface waters also reaching drinking water treatment plants [2,3]. A characteristic structural feature of anabaenopeptins is that they contain a ureido bond connecting the primary amine of a lysine residue with the primary amine of the neighboring amino acid, while the lysine's *ε*-amine forms an amide with the carboxyl group of the C-terminal amino acid to form a five-membered peptide ring. The amino acid building blocks of the core structure can vary, and over 100 anabaenopeptin derivatives have been identified to date [4,5]. Few anabaenopeptins could be tested for their bioactivity so far, but those that were tested showed inhibition of carboxypeptidases A and B [[6], [7], [8], [9]] as well as TAFIa (Activated Thrombin Activatable Fibrinolysis Inhibitor) [10] with IC_50_ values in the low nM concentration range. One obstacle to further explore and quantify biological activities of anabaenopeptins, as with many other natural products, is the lack of available reference material and knowledge about physicochemical properties to assist quantification in purified extracts. To date, over 2400 secondary metabolites have been identified from cyanobacteria [4,5], but their molar extinction coefficients have rarely been reported. Among the more than 300 known microcystins, molar extinction coefficients for 8 variants are known (Table 1), while no values have yet been reported for anabaenopeptins. Herein, we characterized the UV–vis absorbance spectra, molar extinction coefficients and circular dichroism spectra of two prominent anabaenopeptins, i.e., anabaenopeptin A and B.

**Table 1:** Overview of published molar absorbance coefficients of eight microcystin variants and anabaenopeptin A and B.

	Molecular formula	Absorbance max. (nm) in methanol	Molar absorbance coefficient M^−1^cm^−1^)	References
[D-Asp^3^,(*E*)-Dhb^7^] Microcystin RR	C_48_H_73_N_13_O_12_	239	50,400	[11]
Microcystin RR	C_49_H_75_N_13_O_12_	238	39,800	[12]
Microcystin YR	C_52_H_72_N_10_O_13_	231	41,100	[11]
		238 (shoulder)	38,100	[11]
Microcystin LR	C_49_H_74_N_10_O_12_	238	39,800	[12]
		238	36,500	[13]
[D-Asp^3^] Microcystin LR	C_48_H_72_N_10_O_12_	238	31,600	[12]
[Dha^7^] Microcystin LR	C_48_H_72_N_10_O_12_	238	46,800	[12]
[D-Asp^3^,(*E*)-Dhb^7^] Microcystin LR	C_48_H_72_N_10_O_12_	239	31,600	[14]
[D-Asp^3^,(*E*)-Dhb^7^] Microcystin HtyR	C_52_H_72_N_10_O_13_	239	31,600	[14]
Anabaenopeptin A	C_44_H_57_N_7_O_10_	278	4190	*this study*
Anabaenopeptin B	C_41_H_60_N_10_O_9_	278	2300	*this study*

## 3 Data Description

### 3.1 Quantitative NMR

**Fig. 1:**
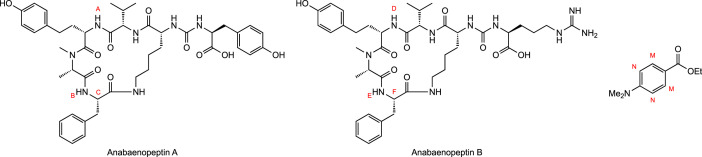
Structures of the analytes anabaenopeptin A and B, and of the internal calibrant ethyl 4-(dimethylamino)benzoate; protons used for calculations are labelled in red.

In order to determine the molar extinction coefficients of anabaenopeptin A and anabaenopeptin B the exact amounts of the extracted samples had to be determined by quantitative NMR (qNMR). Ethyl 4-(dimethylamino)benzoate was used as the internal calibrant (IC) for the measurements performed in DMSO‑*d_6_*. The use of ethyl 4-(dimethylamino)benzoate was prompted by its high solubility in DMSO and the presence of three NMR signals which should not overlap with the target compounds’ signals [15]. The specific protons of the analytes anabaenopeptin A and anabaenopeptin B and the internal calibrant are highlighted in Fig. 1.

**Fig. 2:**
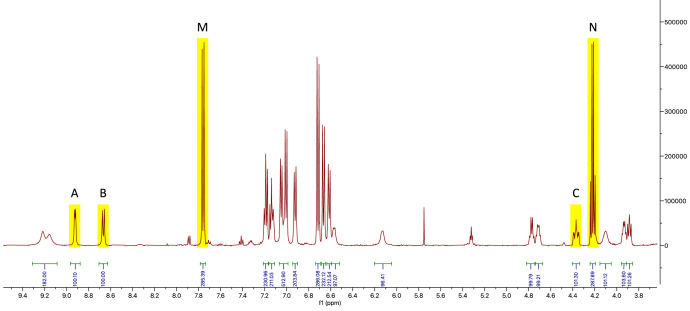
Part of the ^1^H-NMR spectrum of anabaenopeptin A and internal calibrant; signals used for calculations are highlighted in yellow.

**Fig. 3:**
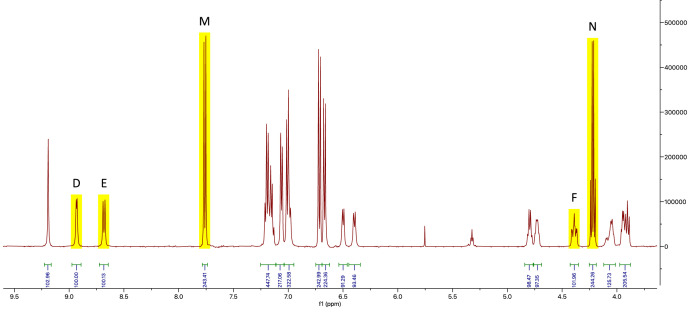
Part of the ^1^H-NMR spectrum of anabaenopeptin B and internal calibrant; signals used for calculations are highlighted in yellow.

In contrast to most other signals in the obtained ^1^H-NMR spectra the ones corresponding to protons A–F of anabaenopeptin A and anabaenopeptin B were clearly separated in their chemical shift resonances from any other signals (Fig. 2, Fig. 3). As anticipated, the same was the case for the two peaks of the internal calibrant labelled as M and N.

The exact amounts of the isolated anabaenopeptin A and anabaenopeptin B samples were calculated according to Eq. (1), with the integral values of the aforementioned peaks and the gravimetrically assessed quantities of the internal calibrant. As shown in Table 2 these were determined to be 1.90 µmol and 2.67 µmol, respectively.

$$n_{A}=\frac{I_{A}}{I_{IC}}\cdot \frac{N_{IC}}{N_{A}}\cdot n_{IC}$$

**Table 2:** Quantification of the two samples containing anabaenopeptin A and B based on the integration of the analyte and internal calibrant signals shown in Fig. 3, Fig. 4.

	Analyte signal (I_A_)	IC signal (I_IC_)	m_IC_ (µg)	n_IC_ (µmol)	n_A_ (µmol)	average n_A_ (µmol)
Anabaenopeptin A	A (100.10)	M (285.39)	524	2.71	1.90	
	B (100.00)	M (285.39)	524	2.71	1.90	1.90
	C (101.30)	N (287.89)	524	2.71	1.91	

Anabaenopeptin B	D (100.00)	M (243.41)	624	3.23	2.66	
	E (100.13)	M (243.41)	624	3.23	2.66	2.67
	F (101.96)	N (244.26)	624	3.23	2.70	

**Equation 1.** Equation used for the quantification of the samples based on the qNMR spectral data: n_A_ = amount of analyte; n_IC_ = amount of the internal calibrant; I_A_ = integral of the analyte signal; I_IC_ = integral of the internal calibrant signal; N_A_ = number of analyte protons; N_IC_ = number of internal calibrant protons.

**Fig. 4:**
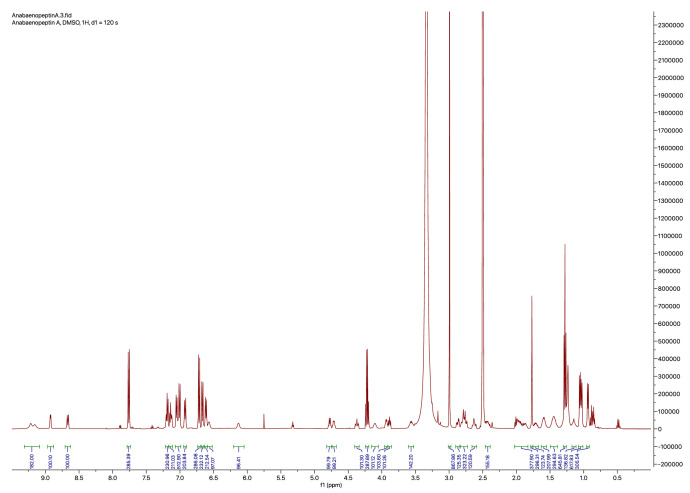
^1^H-NMR spectrum (500 MHz) of anabaenopeptin A and ethyl 4-(dimethylamino)benzoate measured in DMSO‑*d_6_*.

**Fig. 5:**
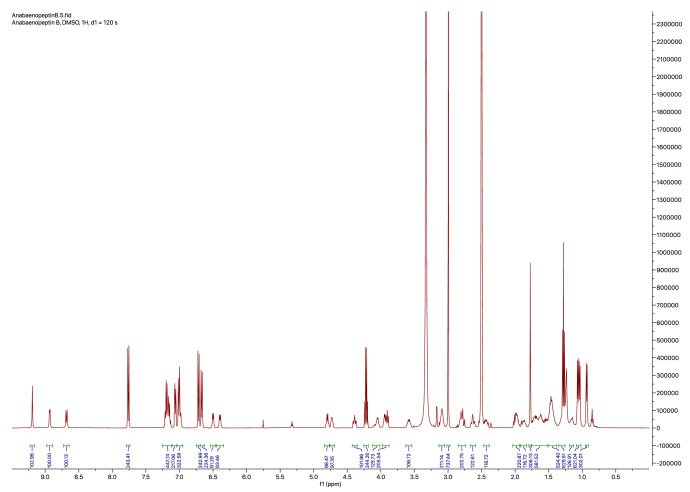
^1^H-NMR spectrum (500 MHz) of anabaenopeptin B and ethyl 4-(dimethylamino)benzoate measured in DMSO‑*d_6_*.

In addition to the quantification of anabaenopeptin A and B, the ^1^H-NMR spectra (Fig. 4, Fig. 5) were also used to confirm their assumed structure. Most signals exactly matched the values reported previously (Table 3, Table 4, [16]). Small deviations in chemical shifts were observed for the exocyclic amino acids (tyrosine in anabaenopeptin A and arginine in anabaenopeptin B) as well as for the two adjacent urea protons.

**Table 3:** Comparison of reported [16] and measured ^1^H-NMR spectral data of anabaenopeptin A; *multiplicity not determined due to overlap with other signals.

Position		Reported [16]				This study			
		ppm		*J*	Hz	ppm		*J*	Hz
Phe	NH	8.65		d	9.0	8.67		d	8.8
	α	4.35		m		4.37		ddd	12.5, 8.8, 3.4
	β	2.75		m		2.77		–*	
		3.25		dd	13.4, 2.6	3.25		–*	
	2,6	7.04	(2H)	d	7.1	7.05	(2H)	d	6.9
	3,5	7.17	(2H)	m		7.19	(2H)	t	7.2
	4	7.12		m		7.13		–*	
MeAla	NMe	1.78	(3H)	s		1.77	(3H)	s	
	α	4.75		q	6.8	4.77		q	6.8
	Me	1.06	(3H)	d	6.8	1.06	(3H)	d	6.6
Hty	NH	8.90		d	4.4	8.92		d	4.6
	α	4.71		m		4.71		m	
	β	1.71		m		1.72		m	
		1.88		m		1.87		–*	
	γ	2.40		m		2.43		m	
		2.59		m		2.63		m	
	2,6	6.99	(2H)	d	8.5	7.01	(2H)	d	8.3
	3,5	6.67	(2H)	d	8.5	6.67	(2H)	d	8.5
	OH	9.18		br. s		9.19		–*	
Val	NH	7.15		–*		7.13		–*	
	α	3.86		m		3.89		dd	8.7, 6.8
	β	1.92		m		1.95		–*	
	Me	0.92	(3H)	d	6.6	0.94	(3H)	d	6.6
	Me’	1.04	(3H)	d	6.6	1.03	(3H)	d	6.7
Lys	α-NH	6.68		–*		6.57		d	7.4
	α	3.97		m		4.10		m	
	β	1.56	(2H)	m		1.60	(2H)	m	
	γ	1.18		m		1.15		m	
		1.28		m		1.28		–*	
	δ	1.42	(2H)	m		1.43	(2H)	m	
	ε	2.78		m		2.77		–*	
		3.52		m		3.56		m	
	ε-NH	7.09		m		7.03		–*	
Tyr	NH	5.95		br. s		6.13		br. s	
	α	3.83		m		3.93		m	
	β	2.78		m		2.77		–*	
		2.86		dd	13.2, 5.6	2.87		dd	13.6, 5.3
	2,6	6.86	(2H)	d	8.3	6.92	(2H)	d	8.0
	3,5	6.54	(2H)	d	8.5	6.61	(2H)	d	7.9
	OH	9.40		br. s		9.19		–*

**Table 4:** Comparison of reported [16] and measured ^1^H-NMR spectral data of anabaenopeptin B; *multiplicity not determined due to overlap with other signals.

Position		Reported [16]				This study			
		ppm		*J*	Hz	ppm		*J*	Hz
Phe	NH	8.62		d	8.5	8.68		d	8.8
	α	4.37		m		4.39		ddd	12.5, 8.9, 3.4
	β	2.78		m		2.80		–*	
		3.30		m		3.30		–*	
	2,6	7.04	(2H)	d	7.1	7.06	(2H)	d	6.9
	3,5	7.17	(2H)	m		7.18	(2H)	–*	
	4	7.12		m		7.15		–*	
MeAla	NMe	1.78	(3H)	s		1.77	(3H)	s	
	α	4.75		q	6.8	4.80		q	6.8
	Me	1.05	(3H)	d	6.8	1.07	(3H)	d	6.5
Hty	NH	8.90		d	4.1	8.93		d	4.7
	α	4.69		m		4.73		m	
	β	1.68		m		1.70		–*	
		1.85		m		1.88		m	
	γ	2.42		m		2.43		m	
		2.61		m		2.63		m	
	2,6	6.99	(2H)	d	8.5	7.01	(2H)	d	8.5
	3,5	6.65	(2H)	d	8.5	6.67	(2H)	d	8.5
	OH	9.18		br. s		9.19		s	
Val	NH	6.99		–*		6.99		–*	
	α	3.86		m		3.94		m	
	β	1.92		m		1.98		–*	
	Me	0.91	(3H)	d	6.6	0.93	(3H)	d	6.5
	Me’	1.02	(3H)	d	6.6	1.04	(3H)	d	6.7
Lys	α-NH	6.60		–*		6.50		d	7.1
	α	3.92		m		4.05		m	
	β	1.57	(2H)	m		1.62	(2H)	–*	
	γ	1.16		m		1.15		m	
		1.29		m		1.29		–*	
	δ	1.40	(2H)	m		1.45	(2H)	–*	
	ε	2.78		m		2.80		–*	
		3.52		m		3.58		m	
	ε-NH	7.05		–*		7.15		–*	
Arg	α-NH	6.14		d	6.1	6.40		d	7.9
	α	3.73		m		3.90		m	
	β	1.52	(2H)	m		1.54		–*	
	γ	1.34				1.45		–*	
		1.40				1.45		–*	
	δ	2.98	(2H)			3.09		m	
	δ-NH	9.45		br. s		–		–	

### 3.2 MS^2^ spectra

**Fig. 6:**
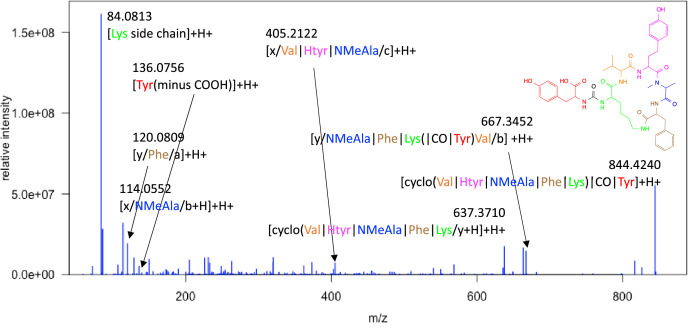
MS^2^ Spectrum of anabaenopeptin A; fragment annotation is indicated with the color-coded building block string [cyclo(Val|Htyr|NMeAla|Phe|Lys)|CO|Tyr].

**Fig. 7:**
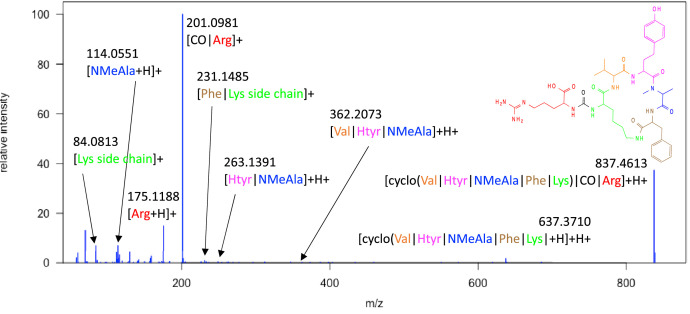
MS^2^ Spectrum of anabaenopeptin B; fragment annotation is indicated with the color-coded building block string [cyclo(Val|Htyr|NMeAla|Phe|Lys)|CO|Arg].

In addition, the structures were also confirmed by tandem mass spectrometry (MS^2^). The molecular ion as well as several key fragments could be identified for anabaenopeptin A (Fig. 6) and anabaenopeptin B (Fig. 7).

### 3.3 UV–vis absorbance spectra

**Fig. 8:**
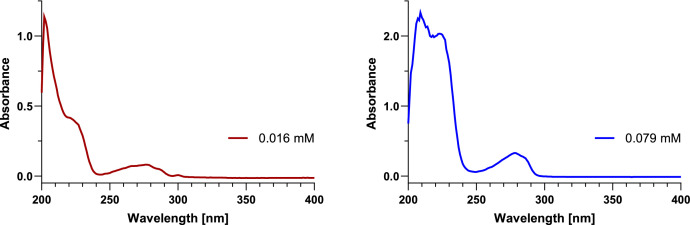
UV–vis absorbance spectra of anabaenopeptin A measured at concentrations of 0.016 mM (left in red) and 0.079 mM (right in blue) in methanol.

**Fig. 9:**
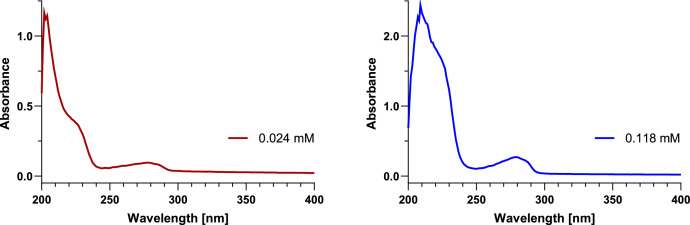
UV–vis absorbance spectra of anabaenopeptin B measured at concentrations of 0.024 mM (left in red) and 0.118 mM (right in blue) in methanol.

UV–vis absorbance spectra of both anabaenopeptin samples were measured at two different concentrations each (approx. 0.10 mM and 0.02 mM). The exact concentrations were determined according to the exact amounts determined by qNMR. The spectra at lower concentrations showed absorbance maxima at 202 nm, shoulders at 219 nm, and broad maxima at 278 nm (Fig. 8, Fig. 9 in red). These maxima at 278 nm became more distinct at higher concentrations (Fig. 8, Fig. 9 in blue) and the corresponding absorbance values for the given concentrations (0.079 mM and 0.118 mM, respectively) were used in the calculations of the molar extinction coefficients (ε).

### 3.4 Molar extinction coefficients (ε)

With the exact concentrations determined by qNMR and the absorbances determined by UV–vis spectroscopy, molar extinction coefficients of 4190 M^−1^cm^−1^ (anabaenopeptin A) and 2300 M^−1^cm^−1^ (anabaenopeptin B) were calculated (Table 5). These values will be useful in future studies to quantify anabaenopeptin A and B in samples of unknown concentration, to verify the concentration of reference standards, and to monitor stability of stock solutions.

**Table 5:** Calculation of the molar extinction coefficient (ε_278_) of anabaenopeptins A and B based on their exact quantity and absorbance.

	Sample amount (µmol)	Concentration (µM)	Absorbance at 278 nm	Molar extinction coefficient (M^−1^cm^−1^)
Anabaenopeptin A	1.90	78.6	0.329	4190
Anabaenopeptin B	2.67	118	0.270	2300

### 3.5 Circular dichroism spectra

**Fig. 10:**
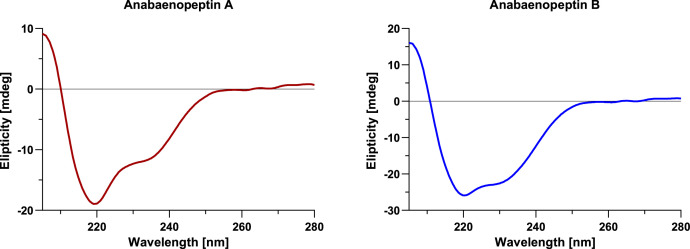
Circular dichroism spectra of anabaenopeptin A (left; median fit in red) and anabaenopeptin B (right; median fit in blue) measured with five repetitions in methanol at concentrations of 0.393 mM and 0.588 mM, respectively.

In addition to the above-mentioned qNMR and UV–vis measurements, circular dichroism analysis was performed. For both anabaenopeptin samples methanolic solutions were prepared and measured with five repetitions (Fig. 10). In the wavelength range below 205 nm only irregular noise could be observed but for both samples all repetitions showed excellent overlap between 205 and 280 nm. The curve shape was similar for both anabaenopeptins, however, this data did not allow to draw any definite conclusions regarding conformational properties of anabaenopeptin A and B.

## 4 Experimental Design, Materials and Methods

### 4.1 Extraction and purification of anabaenopeptins from *Planktothrix rubescens*

The *Planktothrix rubescens* strain used in this study is deposited in the culture collection of Simris Biologics GmbH, Germany, under the accession number CBT929. The strain was cultivated in BG-11 medium at 20 °C [17], illuminated continuously by Sylvania GROLUX fluorescent lamps (50–200 µmol photons m^−2^ s^−1^), and aerated with 0.5–5 % CO_2_ in sterile filtrated air in 20 L polycarbonate carboys. To minimize cell death and lysis, the cultures were harvested weekly and diluted with fresh medium (semi-continuous cultivation to avoid entry into the stationary phase). The biomass was lyophilized and resuspended in 50 % methanol (MeOH, v/v), treated with an ultrasonication rod (Bandelin), and extracted on a shaker for 30 min. After centrifugation, the biomass was subsequently extracted using 80 % MeOH (v/v). The extracts were combined and dried *in vacuo* before being dissolved in 20 % MeOH and fractionated by step-gradient elution on a C18 flash chromatography cartridge (20 %, 40 %, 60 %, 80 %, 100 % MeOH in water). Anabaenopeptins A and B, contained in the fraction eluting with 60 % MeOH, were purified by high-performance liquid chromatography (HPLC) using a Kinetex C18 column (150 × 10 mm, 5 µm, 100 Å, Phenomenex) and a gradient of 10–40 % acetonitrile (MeCN) in H_2_O (0.1 % formic acid) at 5 mL/min over 12 min for anabaenopeptin B and a gradient of 10–47.5 % MeCN in H_2_O (0.1 % formic acid) at 5 mL/min over 12 min for anabaenopeptin A to obtain both compounds with a purity of >99 % based on HPLC-UV and HPLC-MS. Fractions were dried *in vacuo* and stored until further use.

### 4.2 Quantitative NMR

The internal calibrant ethyl 4-(dimethylamino)benzoate was purchased from Sigma-Aldrich and was of *Trace*CERT® grade. Exact gravimetric assessments of the internal calibrant were done on a Mettler Toledo MX5 Microbalance. ^1^HNMR spectra were recorded on a Bruker 500 MHz spectrometer in DMSO‑*d_6_* at 298 K with a recycle delay (d1) of 120 s. Chemical shifts (δ-values) are reported in ppm, spectra were calibrated related to solvent's residual proton chemical shift (DMSO‑*d_5_*, δ = 2.50 ppm), multiplicity is reported as follows: s = singlet, *d* = doublet, *t* = triplet, *m* = multiplet) and coupling constants *J* are reported in Hz.

### 4.3 High resolution mass spectrometry

The high resolution mass spectrometry (HRMS) data were acquired on a Q Exactive Plus mass spectrometer (Thermo Fisher Scientific) equipped with a heated electrospray ionization (ESI) interface coupled to an UltiMate 3000 HPLC system (Thermo Fisher Scientific). The following chromatographic parameters were used: Kinetex C18 column (50 × 2.1 mm, 2.6 µm, 100 Å, Phenomenex), binary gradient from 5 to 100 % MeCN in H_2_O (0.1 % formic acid each) at 0.4 mL/min in 18 min, 100 % MeCN for 2 min. The following parameters were used for mass spectrometry (MS) data acquisition: positive and negative ionization mode, ESI spray voltage of 3.5 kV and −2.5 kV, capillary temperature of 350 °C, sheath gas flow rate of 50 L/min, auxiliary gas flow rate of 12.5 L/min. Full scan accurate mass spectra were acquired from *m/z* 133.4 to 2′000 with a resolution of 35′000 at *m/z* 200, automated gain control (AGC) target 5 × 10^5^, maximum injection time of 120 ms. The MS^2^ were data-dependently (dd-MS^2^) obtained by higher-energy collisional dissociation (HCD) with a stepped collision energy method with 30, 60, and 75 eV (resulting at 55 eV), a resolution of 17′500 at *m/z* 200, an AGC target of 2 × 10^5^, and a maximum injection time of 75 ms. A TopN experiment (*N* = 5, loop count 5) was implemented for triggering the dd-MS^2^ acquisition.

### 4.4 UV–vis spectroscopy

UV–vis absorbance spectra were recorded in analytical grade methanol at room temperature on an Agilent Cary 60 with a scan rate of 300 nm/min and a data interval of 1.00 nm.

### 4.5 Circular dichroism spectroscopy

Circular dichroism spectra were recorded in analytical grade methanol at room temperature on an Applied Photophysics Chirascan™ V100 with five repetitions.

## Limitations

Not applicable.

## Ethics Statement

Authors have read and follow the ethical requirements for publication in Data in Brief and confirm that the presented work did not involve human subjects, animal experiments, or any data from social media platforms.

## CRediT Author Statement

**Till Steiner:** Investigation, Formal analysis, Visualization, Writing – original & editing; **Franziska Schanbacher:** Resources, Writing – review & editing; **Wolfram Lorenzen:** Resources; **Heike Enke**: Resources; **Timo Niedermeyer:** Supervision, Writing – review & editing; **Elisabeth Janssen:** Project administration, Project coordination, Visualization, Writing – original & editing; **Karl Gademann:** Project coordination, Resources, Project administration, Funding acquisition, Writing – editing.

## Data Availability

UV–vis absorbance data, molar extinction coefficients and circular dichroism spectra for the two cyanobacterial metabolites anabaenopeptin A and anabaenopeptin B (Original data) (Zenodo).
